# Definition and Revision of the *Orthrius*-group of genera (Coleoptera, Cleridae, Clerinae)

**DOI:** 10.3897/zookeys.92.1157

**Published:** 2011-04-28

**Authors:** Gerstmeier Roland, Eberle Jonas

**Affiliations:** Technische Universität München, Department für Ökologie und Ökosystemmanagement, Lehrstuhl für Tierökologie, Hans-Carl-von-Carlowitz-Platz 2, 85350 Freising, Germany

**Keywords:** Cleridae, genus-group, synonymy, phylogeny

## Abstract

An “*Orthrius*-group” of genera is proposed, and defined to include *Aphelochroa* Quedenfeldt, 1885; *Caridopus* Schenkling, 1908; *Dozocolletus* Chevrolat, 1842; *Gyponyx* Gorham, 1883; *Languropilus* Pic, 1940; *Orthrius* Gorham, 1876; *Pieleus* Pic, 1940; *Xenorthrius* Gorham, 1892; plus three new genera *Neorthrius* **gen. n.**, *Nonalatus* **gen. n.** and *Pseudoastigmus* **gen. n.** A phylogeny of the 11 constituent *Orthrius*-group genera (analysis of 22 morphological characters using *Clerus* Geoffroy as the out-group taxon was performed with TNT v1.1) is proposed. Four genera are synonymised: *Burgeonus* Pic, 1950, **syn. n.** (with *Aphelochroa* Quedenfeldt, 1885); *Brinckodes* Winkler, 1960, **syn. n.** and *Quasibrinckodes* Winkler, 1960, **syn. n.** (both with *Dozocolletus* Chevrolat, 1842); and *Dedana* Fairmaire, 1888, **syn. n.** (with *Orthrius* Gorham, 1876). The genera *Falsoorthrius* Pic, 1940 and *Mimorthrius* Pic, 1940 are transferred from Clerinae to the subfamily Tillinae.

## Introduction

The checkered beetles (Cleridae and Thanerocleridae) contain approximately 3600 described species, which are classified into seven subfamilies (Lawrence and Newton Jr. 1995) and involve 303 genera. By far, the Clerinae is the most specious subfamily with approximately 45% of the species of the family. Checkered beetles are largely tropical insects with an approximate faunal distribution as follows: 1030 species in the Afrotropics, 840 in the Neotropics, 690 in the Orientalis, 510 in the Australis and 490 in the Palaearctis (Gerstmeier 2000).

The higher classification of the Cleridae has undergone considerable categorical oscillations (Opitz 2002, 2010). Several landmark publications of (Crowson (1955, 1964, 1966, 1970) form the basis for a modern classification of Cleroidea, while some nomenclatural amendments were made by Lawrence and Newton Jr. (1995). More recently, significant contributions dealing with suprageneric taxa include the elevation of Thaneroclerinae (Kolibáč 1992, 2004) and *Metaxina* Broun, to family rank (Kolibáč 1992, 2004), the proposition of two subfamily classifications (Kolibáč 1997, Opitz 2010) plus revisions of the genera and species of Epiphloeinae (Opitz 1997, 2004, 2005, 2006, 2007, 2008a, 2008b, 2008c), the genera of Hydnocerinae (which included a tribal classification for that subfamily)(Kolibáč 1998) and the Australian Korynetinae (Kolibáč 2003). Nevertheless, some discontinuities are obvious and not all changes made at the subfamily-level are universally accepted among cleridologists. From a world viewpoint, much remains to be done with clarification of generic concepts and zoogeographic relationships at supraspecific levels (Opitz 2002). In our opinion, Opitz’s (2010) concept of 12 subfamilies seems to result in the best system.

The Clerinae is the largest of all subfamilies of the Cleridae and the most difficult in which to define generic limits (Chapin 1924). Furthermore, the paucity of clearly defined morphological gaps among these genera renders their generic delimitation very difficult. A paper dealing with genera related to *Clerus* Geoffroy (Gerstmeier 2002) represents an initial step in clarifying generic limits within Clerinae. After an extensive review of Indo-Australian clerid material, a generic concept of clerine genera such as *Clerus* Geoffroy, 1762, *Omadius* Laporte, 1836, and *Stigmatium* Gray, 1832 became apparent and resulted in a preliminary concept of “*Clerus*-series” (Gerstmeier 2002).

A recent revision of the genus *Xenorthrius* Gorham (Gerstmeier and Eberle 2010) represents besides Mawdsley’s (1994) revision of the genus *Aphelochroa* the second in a series of papers dealing with the genera of a so-called *“Orthrius*-group”. In the *Xenorthrius* revison 11 species were transferred from *Orthrius* to *Xenorthrius*, and 22 new species were described, so that the genus *Xenorthrius* now includes 50 species (from 20 species formerly listed in Corporaal 1950). The aim of the present paper is to define the characters for a generic group, to determine those genera constituting the *Orthrius* group and examine the relationships among those genera. The following genera have been taken into consideration: *Aphelochroa* Quedenfeldt, 1885, *Caridopus* Schenkling, 1908, *Dozocolletus* Chevrolat, 1842, *Gyponyx* Gorham, 1883, *Languropilus* Pic, 1940, *Neorthrius* gen. n., *Orthrius* Gorham, 1876, *Pieleus* Pic, 1940, *Nonalatus* gen. n., *Pseudoastigmus* gen. n., *Xenorthrius* Gorham, 1892, *Falsoorthrius* Pic, 1940 and *Mimorthrius* Pic, 1940 (during this study, the latter two genera were discovered to belong to the subfamily Tillinae).

## Historic overview

Gorham (1876) described the genus *Orthrius* for *Orthrius cylindricus* and noticed the relationship to *Thanasimus*, and, on the basis of the tarsal structure, to *Clerus*. Seven years later, the same author (Gorham 1883) established the genus *Gyponyx* and mentioned its relationship to *Thanasimus* and *Axina*. Chevrolat (1842) described the species “*oblongus*”, drawing attention to its flightlessness and established the genus *Dozocolletus*, without a generic diagnosis; a diagnosis was given later by Lacordaire (1857). Quedenfeldt (1885) described the genus *Aphelochroa* (with *Aphelochroa carneipennis* as type species) comparing it with *Opilo* and *Natalis*. Later, Gorham (1892) established the new genus *Xenorthrius* for three new species (*Xenorthrius balteatus*, *Xenorthrius mouhoti* and *Xenorthrius subfasciatus*). For another two wingless species Schenkling (1908) erected the genus *Caridopus* and in the same publication, described the species *Apteroclerus brevis* from the Kilimanjaro, though with reservations about its generic placement. In two different publications (Pic (1940a, 1940b) respectively described the genera *Languropilus* and *Pieleus*, while in an earlier paper (Pic 1933), he had expressed his view that the flightless *Astigmus pygidialis* differs greatly from all other *Astigmus* species.

## Material and methods

### Abbreviations

A Antennomere

CuA2 Cubitus anterior 2

MNHN Museum National d’Histoire Naturelle, Paris, France

MRAC Musée Royal de l’Afrique Central, Tervuren, Belgium

MSNG Museo Civico di Storia Naturale “Giacomo Doria”, Genova

MZLU Museum of Zoology, Lund University, Sweden

RGCM Roland Gerstmeier Collection, Munich (deposited in the collection of the Technical University Munich, Animal Ecology), Germany

r3, r4 Radial cross vein 3 and 4

RP2 Radius posterior 2

SDEI Senckenberg Deutsches Entomologisches Institut, Müncheberg, Germany

T Tarsomere

### Cladistic analysis

23 characters with their respective states (Tab. 1) were analysed. Character polarity was determined by the outgroup method (Nixon and Carpenter 1993); no ancestral states were forced. The genus *Clerus* Geoffroy, 1762, was considered the outgroup taxon. The data matrix (Tab. 2) was analysed with the Willi Hennig Society edition of TNT 1.1 from September 2009 (Goloboff et al. 2003, 2008). To receive an exact solution, every possible tree was computed by using the “implicit enumeration” routine.

For characters with more than one state per genus, multiple character states were used; they appear enclosed by square brackets in the matrix. Characters that were ambiguous, or missing in the available specimen, appear as a question mark. All characters were chosen to be nonadditive and none were weighted. Implied weighting was also turned off. The species were sorted alphabetically within the input file.

## Diagnosis

**Figures 1–10.**
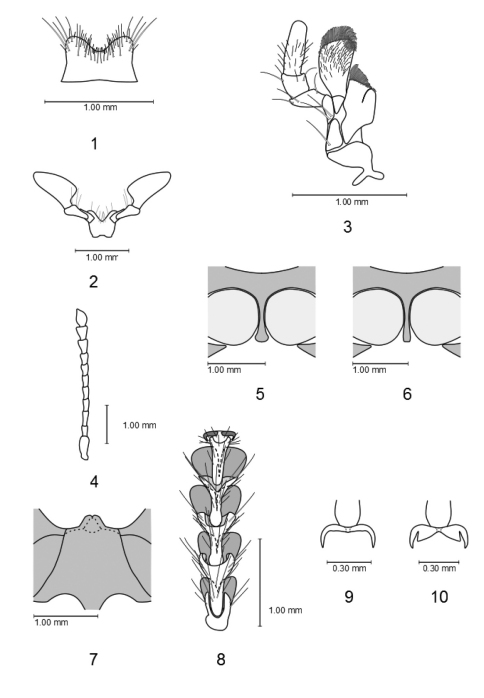
**1–4** Labrum, labium, maxille and antenna of *Orthrius sepulcralis* **5** Pro-intercoxal process of *Xenorthrius robustus* **6** Pro-intercoxal process of *Orthrius sepulcralis* **7** Anterior mesosternal process of *Xenorthrius robustus* **8–9** Tarsus and claws of *Orthrius sepulcralis* **10** Claws of *Xenorthrius robustus*.

Species of the *Orthrius*-group are readily distinguished from other Clerinae by the presence of the following characters (in combination):

– Eyes distinct, more or less protruding laterally, coarsely facetted

– Eyes separated by more than one eye width

– Labrum bilobed to broadly V-shaped (Fig. 1)

– Terminal segment of labial palpi securiform (Fig. 2)

– Terminal segment of maxillary palpi cylindrical (to digitiform) (Fig. 3)

– Antennal flagellum more or less filiform (Fig. 4)

– Antennomere 2 shorter than antennomere 3 (except *Languropilus*)

– Procoxal cavities broadly open posteriorly (Figs 5, 6)

– Pro-intercoxal process not (or only slightly) dilated distally (Figs 5, 6)

– Metendosternites without anterior tendons, furcal arms distinct, furcal laminae mostly distinct, furcal stalks mostly of normal length or very short in wingless genera (Figs 11–20)

– Elytra without sharply-defined basal margin

– Typical wing venation (if winged), with open wedge cell, r3, r4 and CuA2 (except *Pieleus*) present, RP2 absent (Figs 21–28)

– Pro- and meso-tarsi each with four pulvilli (number of metatarsal pulvilli variable) (Fig. 8)

– Hind tarsi: T2<T3 + T4 (Tarsomere 2 smaller than tarsomeres 3 and 4 together)

– Spiculae of spicular fork more or less dilated (Figs 29–37)

## Key to genera

**Table:** 

1	Pronotum with six gibbosities on disc	*Caridopus*
–	Pronotum without six gibbosities on disc	2
2	Wingless species	3
–	Species with wings	5
3	Antennomeres of flagellum from A4 dilated, antennal club absent	4
–	Flagellum filiform, antennal club with 3 antennomeres	*Dozocolletus*
4	Tarsal pulvillar formula 4-4-2	*Nonalatus* gen. n.
–	Tarsal pulvillar formula 4-4-3	*Pseudoastigmus* gen. n.
5	Claws dentate (Fig. 10)	6
–	Claws simple (Fig. 9)	7
6	Tibial spur formula 1-1-2, elytral punctation not arranged into 10 striae	*Pieleus*
–	Tibial spur formula 1-2-2, elytral punctation arranged into 10 striae	*Xenorthrius*
7	Anterior mesosternal process present (Fig. 7), tarsal pulvillar formula 4-4-4	*Gyponyx*
–	Anterior mesosternal process absent, tarsal pulvillar formula 4-4-3	8
8	Tibial carinae absent	*Languropilus*
–	Tibial carinae present	9
9	Eyes weakly emarginate	10
–	Eyes conspicuously emarginate	*Neorthrius* gen. n.
10	Tibial spur formula 1-2-2	*Aphelochroa*
–	Tibial spur formula 0-1-1	*Orthrius*

## Description of genera

### Aphelochroa

Quedenfeldt, 1885

http://species-id.net/wiki/Aphelochroa

**Figures 11–20.**
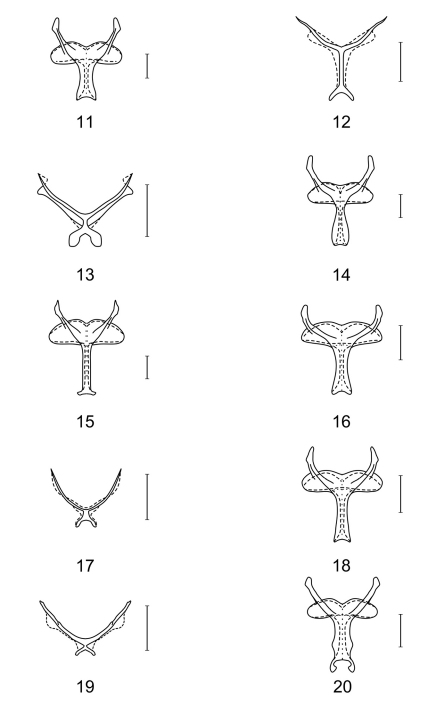
Metendosternites of **11** *Aphelochroa* sp. **12** *Caridopus* sp. **13** *Dozocolletus discophorus* **14** *Gyponyx* sp. **15** *Languropilus fortipes* **16** *Neorthrius* sp. **17** *Nonalatus brevis* **18** *Orthrius sepulcralis* **19** *Pseudoastigmus pygidialis* **20** *Xenorthrius loricus*. Scale bars 0.5mm.

Figs 11 21 29 38 47 58

Burgeonus Pic, 1950 syn. n.; Pic 1950: 158.

#### Type species:

*Aphelochroa carneipennis* Quedenfeldt, 1885. Quedenfeldt 1885: 267; Kraatz 1899: 86; Schenkling 1902: 326; Schenkling 1903: 29, 57; Mawdsley 1994: 128; Mawdsley and Sithole 2010: 1.

#### Distribution:

Aethiopian region.

#### Material examined:

*Aphelochroa sanguinea* (Thomson, 1857), Kenya, Voi, Sagala Region, 12.1991, leg. K. Werner. *Aphelochroa sanguinalis* (Westwood, 1852), Congo, VIII.1959, Albertville. *Aphelochroa fulva* Kraatz, 1899, Kenya, Meru Distr., Materi (Mitunguu), mt. 800, R. Mourglia legit; and several other specimens of this genus (all RGCM). *Burgeonus freynei* Pic, 1950 (Holotype), Coll. Mus. Congo, Lulua: Luashi, XI-1938, F. Freyne; R. DET., X., 5621; desiré; Burgeonus freynei n sp [handwritten by Pic](MRAC).

#### Description

##### Head:

Eyes strongly protruding, only slightly emarginate at antennal insertion; interocular space more than one eye width; gular sutures converging, gular process broad; A1 large, stout, almost twice as long as A2, A2 shorter than A3, A3-A8 filiform, antennomeres becoming shorter, A9 dilated distally, A10 broader than long, A11 sub-ovate, apical third pinched, terminal three antennomeres forming a loose club.

##### Thorax:

Proepimeron short, not acute; anterior mesosternal process absent; proepimeron short; metendosternite with normal furcal stalk, short, normal furcal arms and very slightly emarginate stalk base (Fig. 11). Elytra long, subparallel, broadest behind middle, apices broadly rounded, elytral punctation not arranged into striae.

##### Legs:

Of normal size, stout; tarsal pulvillar formula 4-4-3, tibial spur formula 1-2-2; tibiae with longitudinal carinae; claws simple.

##### Abdomen:

Apical margin of male ventrite 6 distinctly emarginate (Fig. 47); tegmen slender, tapering to a curved acumination distally, phallic struts acuminate, not fused, phallobasic apodeme slightly dilated distally (Fig. 38).

### Caridopus

Schenkling, 1908

http://species-id.net/wiki/Caridopus

Figs 12 22 30 39 48 59

#### Type species:

*Caridopus monstruosus* Schenkling, 1908. Schenkling 1908: 71.

#### Distribution:

Aethiopian region.

#### Material examined:

*Caridopus monstruosus* (Type), Kilimandj., Sjöstedt; Kibonoto, kulturz.; 30. April; Caridopus monstruosus, Typus! (NRM). *Caridopus affinis* Schenkling, 1908 (Type), Meru, Regenwald; Meru, Sjöstedt; Caridopus affinis Schklg., Typus! (NRM).

#### Description

##### Head:

Eyes strongly protruding, only slightly emarginate at antennal insertion; interocular space more than 1.5 eye widths; gular sutures converging, gular process broad; antennae long, A2 shorter than A3, A3-A8 filiform, antennomeres becoming shorter, A9 and especially A10 dilated distally, A10 shorter than A9, A11 sub-ovate, apical third pinched, without club.

##### Thorax:

Conspicuously longer than broad, with six gibbosities on disc; pro-intercoxal process narrow, linear; proepimeron short, acute to slightly rounded; anterior mesosternal process present; metendosternite with normal furcal stalk length, furcal arms acute distally, stalk base conspicuously emarginate (Fig. 12). Elytra compact (broadest behind middle), conspicuously constricted at base and strongly dilated apically in wingless species, apices broadly rounded, elytral punctation arranged into ten more or less regular striae; wingless or with hindwings.

##### Legs:

Long, very stout, femora conspicuously thickened; tarsal pulvillar formula 4-4-4, tibial spur formula 1-2-2; tibiae without longitudinal carinae; claws simple.

##### Abdomen:

Apical margin of male ventrite 6 deeply emarginate (Fig. 48); phallobasic struts fused, phallic struts very broad, phallobasic apodeme strongly dilated distally (Fig. 39).

**Figures 21–28.**
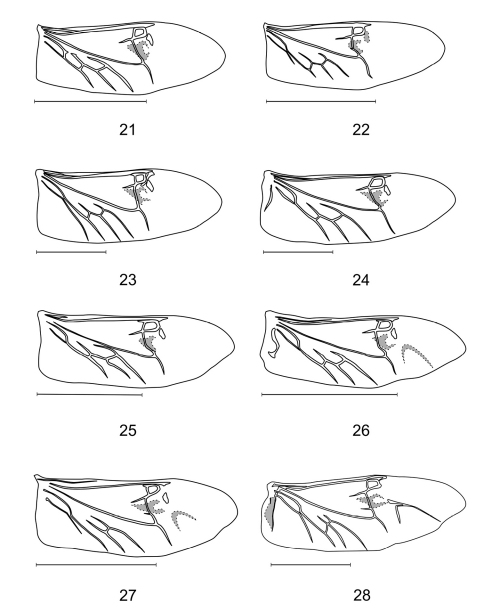
Wings of **21** *Aphelochroa* sp. **22** *Caridopus* sp. **23** *Gyponyx* sp. **24** *Languropilus* sp. **25** *Neorthrius* sp. **26** *Orthrius* sp. **27** *Pieleus* sp. **28** *Xenorthrius* sp. Scale bars 5mm.

### Dozocolletus

Chevrolat, 1842

http://species-id.net/wiki/Dozocolletus

Figs 13 31 40 49 60

Brinckodes Winkler, 1960 syn. n.; Winkler 1960: 130.
Quasibrinckodes Winkler, 1960 syn. n.; Winkler 1960: 134.

#### Type species:

*Dozocolletus oblongus* Chevrolat, 1842. Chevrolat 1842: 278; Lacordaire 1857: 442; Schenkling 1903: 28, 38.

#### Distribution:

Aethiopian region (southern Africa).

#### Material examined:

*Dozocolletus discophorus* (Boheman, 1851)(Type), Caffraria, J. Wahlb, Type. *Dozocolletus puberulus* (Boheman, 1851)(Type), Caffraria, J. Wahlb, Type. *Dozocolletus oblongus* Chevrolat, 1842, Pretoria, 2.XII.1963, leg. A.L. Capener (all NRM). *Brinckodes apterus* Winkler, 1960 (Holotype and two Paratypes), S. Afr. Transvaal, 16 miles NE of Pretoria, Oct.-Nov. 1954, G. Rudebeck; Brinckodes apterus n.g., n.sp., Det. J.R. Winkler, 1959. *Brinckodes apterus* ab. *ater* Winkler, 1960 (Holotype), S. Afr. Transvaal, 16 miles NE of Pretoria, Oct.-Nov. 1954, G. Rudebeck; Brinckodes apterus n.g., n.sp., n.ab., Det. J.R. Winkler 1959. *Quasibrinckodes pictus* Winkler, 1960 (Holotype), 8200 ft.; S. Afr. Cape Prov., Drakensbergen, 8 miles ENE Rhodes, 10.III.51, No 223; Swedish South Africa Expedition, 1950–1951, Brinck - Rudebeck; Quasibrinckodes pictus n.g., n.sp., Det. J.R. Winkler 1959, Holotypus (all MZLU).

#### Description

##### Head:

Eyes protruding, very slightly emarginate at antennal insertion; interocular space two to three eye widths; gular sutures converging, gular process broad; antennae long, A1 large, stout, almost twice as long as A2, A2 shorter than A3, A3-A8 filiform, antennomeres becoming shorter, A9 short, transverse, A10 larger than A9, transverse, A11 approximately equal in length to A9+A10, sub-ovate, apical half pinched, terminal three antennomeres forming a distinct club.

##### Thorax:

Pronotum conspicuously constricted towards base, without transverse impression, proepimeron short to medium-sized, not acute; anterior mesosternal process present, broadly bent, proepimeron broad, short; metendosternite with very short furcal stalk, stalk base broad, with a deep emargination, furcal arms long, acute distally (Fig. 13). Elytra short, elytral base strongly constricted, broadest behind middle, apices rounded, elytral punctation arranged into ten striae; wingless.

##### Legs:

Relatively short, stout; femora conspicuously thickened (especially profemora); tarsal pulvillar formula 4-4-4, tibial spur formula 2-2-2; tibiae with longitudinal carinae; claws simple, stout.

##### Abdomen:

Apical margin of male ventrite 6 not emarginate (Fig. 49); tegmen relatively broad, phallobasic struts fused, phallic struts broad, dilated distally, phallobasic apodeme not dilated distally (Fig. 40).

**Figures 29–37.**
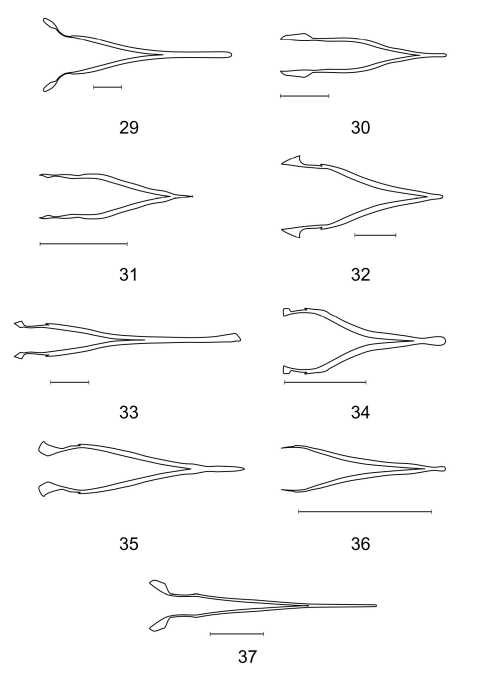
Spicular forks of **29** *Aphelochroa* sp. **30** *Caridopus monstruosus* **31** *Dozocolletus discophorus* **32** *Gyponyx* sp. **33** *Neorthrius* sp. **34** Nonalatus brevis **35** *Orthrius sepulcralis* **36** *Pseudoastigmus pygidialis* **37** *Xenorthrius simplex*. Scale bars 0.5mm.

### Gyponyx

Gorham, 1883

http://species-id.net/wiki/Gyponyx

Figs 14 23 32 41 50 61

#### Type species:

*Notoxus chinensis* Fabricius, 1794. Gorham 1883: 604; Schenkling 1900: 14; Schenkling 1903: 29, 45; Schenkling 1907: 199.

#### Distribution:

Aethiopian region.

#### Material examined:

*Gyponyx apicalis* (Chevrolat, 1842), Südafrika, SE 3130AA, Umtanvuma 3.1.1989, leg. T. Beyers; *Gyponyx signifer* (Boheman, 1851), Tanzania, Nufindi Dist., Nafinga 1000m, 21.11.-4.12.1989, leg. R. Mourglia; and several further specimens of this genus (all RGCM).

#### Description

##### Head:

Eyes strongly protruding, broadly but not deeply emarginate at antennal insertion; interocular space more than 1.5 eye widths; gular sutures converging, gular process broad; A1 large, stout, almost two times longer than A2, A2 shorter than A3, A3-A6 filiform, A7-A10 slightly dilated distally, antennomeres becoming shorter, A11 sub-ovate, apical third pinched, without club.

##### Thorax:

Proepimeron medium-sized, more rounded than acute; anterior mesosternal process present; metendosternite with normal furcal stalk, short, normal furcal arms and very slightly emarginate stalk base (Fig. 14). Elytra long, subparallel, strongly dilated apically (broadest behind middle), apices broadly rounded, elytral punctation arranged into ten more or less regular striae.

##### Legs:

Of normal size; tarsal pulvillar formula 4-4-4, tibial spur formula 2-2-2; tibiae with longitudinal carinae; claws simple.

##### Abdomen:

Apical margin of male ventrite 6 very slightly emarginate (Fig. 50); tegmen broad, phallobasic struts fused, phallic struts and phallobasic apodeme broad, but not conspicuously dilated distally (Fig. 41).

### Languropilus

Pic, 1940

http://species-id.net/wiki/Languropilus

Figs 15 24 51 62

#### Type species:

*Languropilus fortipes* Pic, 1940. Pic 1940a: 3.

#### Distribution:

Aethiopian region (East Africa).

#### Material examined:

*Languropilus fortipes* (females), Tanzania, Shinyanga Prov., Serengeti Sopa L., 19.XI.93, LF., Heiss (RGCM).

#### Description

##### Head:

With weakly protruding eyes, only very slightly emarginate at antennal insertion; interocular space about two times one eye width; gular sutures long, converging, gular process broad; antennae short, A1 large, stout, almost two times longer than A2, A2=A3 or A2>A3, A3-A8 filiform, antennomeres becoming shorter, A8 almost spherical, A9 and A10 transverse, A11 ovate, terminal three antennomeres forming a distinct club.

##### Thorax:

Proepimeron medium-sized, more rounded than acute; anterior mesosternal process absent; metendosternite with normal furcal stalk length, stalk slender, base almost straight, furcal arms of more or less normal length, acute distally (Fig. 15). Elytra long, broadest behind middle, apices broadly rounded, elytral punctation arranged into ten striae.

##### Legs:

Of normal size, stout; tarsal pulvillar formula 4-4-3, tibial spur formula 1-2-2; tibiae without longitudinal carinae; claws simple.

**Figures 38–46.**
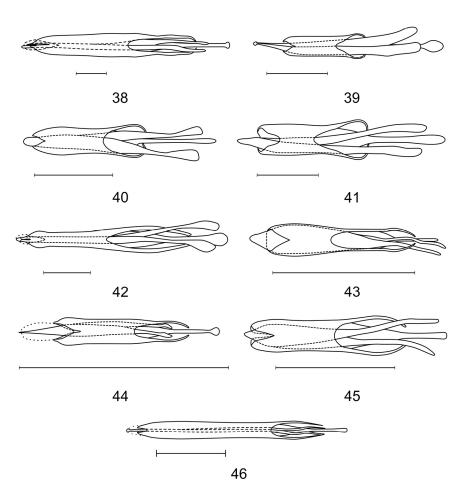
Aedeagi of **38** *Aphelochroa* sp. **39** *Caridopus* sp. **40** *Dozocolletus discophorus* **41** *Gyponyx* sp. **42** *Neorthrius* sp. **43** *Nonalatus brevis* **44** *Orthrius sepulcralis* **45** *Pseudoastigmus pygidialis* **46** *Xenorthrius simplex*. Scale bars 1mm.

### Neorthrius

Gerstmeier & Eberle gen. n.

urn:lsid:zoobank.org:act:37980052-1760-48A2-8AA8-F677612AA8AE

http://species-id.net/wiki/Neorthrius

Figs 16 25 33 42 52 63

#### Type species:

*Neorthrius monticola* Schenkling, 1906 Schenkling 1906: 267.

#### Distribution:

Indo-Australian region.

#### Material examined:

*Neorthrius monticola* (Holotype), Kina-Balu-Geb., 1500m, Coll. Waterstrad; Schenkling det (SDEI); and several unidentified specimens of this genus.

#### Description

##### Head:

Eyes strongly protruding, conspicuously emarginate at antennal insertion; interocular space at least more than one eye width; gular sutures converging, gular process broad, compact, only slightly emarginate at middle; antennae long, A1 about two times longer than A2, A2 shorter than A3, A3-A8 filiform, A9 and A10 slightly dilated distally, A3-A5 more or less equal in length, A6-A8 becoming shorter, A11 sub-ovate, apical half pinched, sometimes without club, sometimes terminal three antennomeres forming a loose club.

##### Thorax:

Proepimeron short to medium-sized, more rounded than acute; anterior mesosternal process absent; metendosternite with normal furcal stalk length, furcal arms normal, stalk base slightly emarginate (Fig. 16). Elytra long, subparallel, sometimes constricted apically, apices rounded separately, elytral punctation arranged into ten striae.

##### Legs:

Of normal size, sometimes with thickened femora; tarsal pulvillar formula 4-4-3, tibial spur formula 1-2-2; tibiae with longitudinal carinae; claws simple.

##### Abdomen:

Apical margin of male ventrite 6 sometimes deeply emarginate (Fig. 52); phallobasic struts not fused, phallic struts and phallobasic apodeme dilated distally (Fig. 42).

**Figures 47–57.**
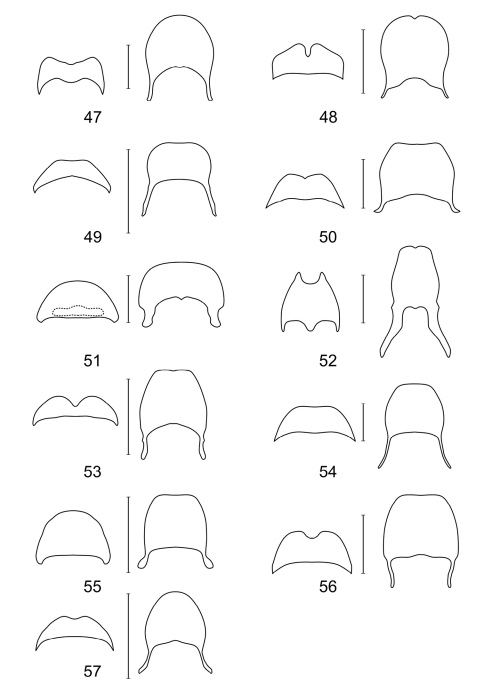
Terminal abdominal segments of **47** *Aphelochroa* sp. **48** *Caridopus* sp. **49** *Dozocolletus discophorus* **50** *Gyponyx* sp. **51** *Languropilus fortipes* **52** *Neorthrius* sp. **53** *Nonalatus brevis* **54** *Orthrius sepulcralis* **55** *Pieleus irregularis* **56** *Pseudoastigmus pygidialis* **57** *Xenorthrius simplex*. Scale bars 1mm.

### Nonalatus

Gerstmeier gen. n.

urn:lsid:zoobank.org:act:4331C030-7A93-4F7D-A6F1-D896965CF99D

http://species-id.net/wiki/Neorthrius

Figs 17 34 43 53 64

#### Type species:

*Apteroclerus brevis* Schenkling, 1908, comb. n. Schenkling 1908: 71.

#### Distribution:

Aethiopian region (Kilimanjaro).

#### Material examined:

*Apteroclerus brevis* (Type), Kilimandj., Sjöstedt; Kiboscho, 3’-4000m; 15. febr.; Bärgs-ängarne; Typus; Bergwiesen, Ericinella-Region, In den trockenen Blumenständen von Lobelia deekeni (NRM).

#### Description

##### Head:

Eyes protruding, emarginate at antennal insertion; interocular space two to three eye widths; gular sutures strongly diverging, gular process broad; antennae long, A2 shorter than A3, from A4 slightly dilated apically, A3-A7 becoming shorter, A9 and A10 more or less equal in length, A11 longer than A10, A11 sub-ovate, apical third pinched, without club.

##### Thorax:

Proepimeron short, not acute; anterior mesosternal process present, broadly bent; metendosternite with very short furcal stalk, stalk base deeply emarginate, furcal arms acute distally (Fig. 17). Elytra ovate, short, compact, strongly constricted at base and towards apex, broadest behind middle, apices broadly rounded, elytral punctation arranged into ten irregular striae; wingless.

##### Legs:

Relatively long, stout; tarsal pulvillar formula 4-4-2, tibial spur formula 1-2-2; tibiae without longitudinal carinae; claws simple, with a very small, acute basal denticle.

##### Abdomen:

Apical margin of male ventrite 6 distinctly emarginate (Fig. 53); tegmen very broad, parameres expanded laterally, tapering to an acumination distally, phallobasic struts not fused, phallic struts and phallobasic apodeme not dilated distally (Fig. 43).

**Figures 58–63.**
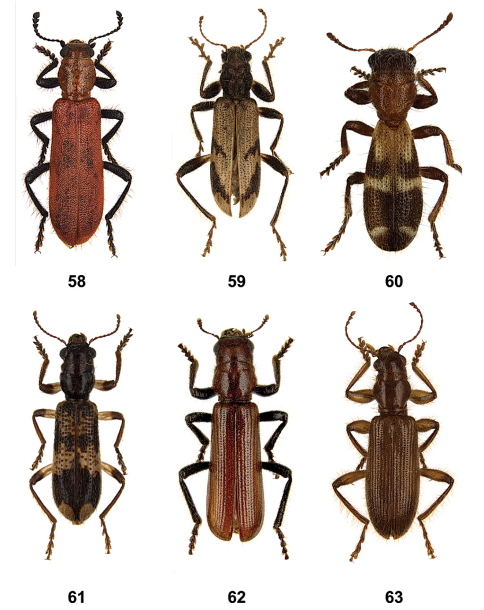
Habitus of **58** *Aphelochroa* sp. **59** *Caridopus* sp. **60** *Dozocolletus discophorus* **61** *Gyponyx* sp. **62** *Languropilus fortipes* **63** *Neorthrius* sp.

### Orthrius

Gorham, 1876

http://species-id.net/wiki/Orthrius

Figs 1–4, 6, 8–9 18 26 35 44 54 65

Dedana Fairmaire, 1888, syn. n.; Fairmaire 1888: 26; Schenkling 1903: 4, 23.

#### Type species:

*Orthrius cylindricus* Gorham, 1876. Gorham 1876: 74.

#### Distribution:

Indo-Australian region.

#### Material examined:

*Orthrius cylindricus* (Type), NSW; Orthrius Gorh., cylindricus G., Type; Museum Paris, Coll. Gorham, 1914 (MNHN); and several other specimens of this genus. *Dedana rufodorsata* Fairmaire, 1888 (Type), Fokien; Dedana rufodorsata Fairm.; ExMusaeo Arm. David, 1900 (MNHN).

#### Description

**Head:** Eyes strongly protruding, only slightly emarginate at antennal insertion; interocular space more than one eye width; gular sutures converging, gular process broad; antennae long, A2 shorter than A3, A2-A8 filiform, A10 broadest, A11 sub-ovate, apical half pinched, terminal three antennomeres forming a more or less conspicuous club.

**Thorax:** Proepimeron short to medium-sized, not acute; anterior mesosternal process absent; metendosternite with normal furcal stalk length, furcal arms normal, stalk base very slightly emarginate (Fig. 18). Elytra long, subparallel, sometimes dilated apically (broadest behind middle), apices rounded, elytral punctation not arranged into striae.

**Legs:** Long, especially profemora intermediately to strongly thickened; tarsal pulvillar formula 4-4-3, tibial spur formula 0-1-1; tibiae with longitudinal carinae; claws simple.

**Abdomen:** Apical margin of male ventrite 6 straight or slightly emarginate (Fig. 54); tegmen relatively broad, parameres expanded laterally, tapering to a curved acumination distally, phallobasic struts not fused, phallobasic apodeme dilated distally (Fig. 44).

**Figures 64–68.**
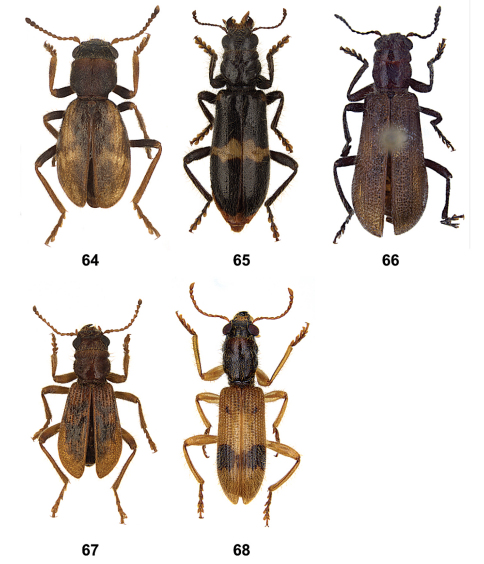
Habitus of **64** *Nonalatus brevis* **65** *Orthrius sepulcralis* **66** *Pieleus irregularis* **67** *Pseudoastigmus pygidialis* **68** *Xenorthrius mouhoti*.

### Pieleus

Pic, 1940

http://species-id.net/wiki/Pieleus

Figs 27 55 66

#### Type species:

*Pieleus irregularis* Pic, 1940. Pic 1940b: 4.

#### Distribution:

China.

#### Material examined:

*Pieleus irregularis* (Type female), T’ienmu Shan, Musée Heude; 20.VII.36, O. Piel, coll.; Orthrius irregularis mihi [handwritten by Pic](MNHN).

#### Description

##### Head:

Eyes strongly protruding, conspicuously emarginate at antennal insertion; interocular space about 1.5 eye widths; gular sutures diverging, gular process broad; antennae short, A1 more than two times longer than A2, A2 shorter than A3, A2-A6 filiform, antennomeres becoming shorter, A7 shorter than A6, slightly dilated distally, A11 sub-ovate, apical third pinched, terminal three antennomeres forming a loose club.

##### Thorax:

Proepimeron medium-sized, slightly rounded; anterior mesosternal process present; metendosternite missing. Elytra compact, strongly dilated apically (broadest behind middle), apices broadly rounded, elytral punctation not arranged into striae; without CuA2 in hindwings (Fig. 27).

##### Legs:

Of normal size; tarsal pulvillar formula 4-4-4, tibial spur formula 1-1-2; tibiae without longitudinal carinae; claws with basal denticle.

### Pseudoastigmus

Eberle gen. n.

urn:lsid:zoobank.org:act:56520F10-5440-4188-ACCE-CBA847869133

http://species-id.net/wiki/Pseudoastigmus

Figs 19 36 45 56 67

#### Type species:

*Astigmus pygidialis* Pic, 1933, comb. n. Pic 1933: 257.

#### Distribution:

Aethiopian region (Ruwenzori).

#### Material examined:

*Astigmus pygidialis* (Syntype), Musée du Congo, Ruwenzori (4200m), VII-1932, L. Burgeon; type; Stigmatium (Astigmus) pygidiale n sp [handwritten by Pic], and four additional syntypes (MRAC).

#### Description

##### Head:

Eyes strongly protruding, conspicuously emarginate at antennal insertion; interocular space about two eye widths; gular sutures subparallel to slightly diverging, gular process of medium width; antennae long, A2 shorter than A3, from A4 onwards slightly dilated distally, A11 sub-ovate, apical half pinched, without club.

##### Thorax:

Proepimeron very short, not acute; anterior mesosternal process present; metendosternite with very short furcal stalk length, furcal arms acute distally, stalk base conspicuously emarginate (Fig. 19). Elytra short, compact, dilated apically (broadest behind middle), apices broadly rounded, elytral punctation arranged into more or less regular ten striae; wingless.

##### Legs:

Long, stout; tarsal pulvillar formula 4-4-3, tibial spur formula 1-2-2; tibiae without longitudinal carinae; claws with basal denticle.

##### Abdomen:

Apical margin of male ventrite 6 deeply emarginate (Fig. 56); tegmen relatively broad, tapering to a curved acumination distally, phallobasic struts not fused, phallic struts and phallobasic apodeme not dilated distally (Fig. 45).

### Xenorthrius

Gorham, 1892

http://species-id.net/wiki/Xenorthrius

Figs 5, 7, 10 20 28 37 46 57 68

#### Type species:

*Xenorthrius mouhoti* Gorham, 1892. Gorham 1892: 733, 1893: 575; Schenkling 1903: 46–47.

#### Distribution:

Indo-Australian and Palaearctic region.

#### Material examined:

*Xenorthrius mouhoti*, Lectotype (MSNG), Paralectotypes, and additional species (see Gerstmeier and Eberle 2010).

#### Description

##### Head:

Eyes strongly protruding, conspicuously emarginate at antennal insertion; interocular space larger than one eye width; gular sutures subparallel to divergent, gular process varying in width, from narrow to broad; antennal length interspecifically variable and sometimes sexually dimorphic (longer in males), A2 shorter than A3, A3-A8 more or less filiform, A10 broader than long, A11 sub-ovate, apical half pinched, mostly without club, sometimes terminal three antennomeres forming a loose club.

##### Thorax:

Proepimeron medium-sized, more or less acute; anterior mesosternal process present, with a subtriangular sulcus in the middle (Fig. 7); metendosternite with normal furcal stalk length, furcal arms broad, apically dilated, stalk base very slightly to deeply emarginate (Fig. 20). Elytra subparallel, sometimes broadest behind middle, apices rounded (most species), strongly dehiscent (*Xenorthrius prolongatus* and *Xenorthrius furcalis*), or dentate (*Xenorthrius truncatus* and *Xenorthrius scordalus*); elytral punctation arranged into ten striae.

##### Legs:

Mostly relatively short; tarsal pulvillar formula 4-4-4, tibial spur formula 1-2-2; tibiae with or without longitudinal carinae; claws with pronounced basal denticle (Fig. 10).

##### Abdomen:

Apical margin of male ventrite 6 more or less distinctly emarginate (Fig. 57); tegmen mostly elongate, cross-section subrectangular; phallobasic struts not fused, phallic struts acute, phallobasic apodeme not dilated distally (Fig. 46).

## Discussion of cladistic results

**Figure 69.**
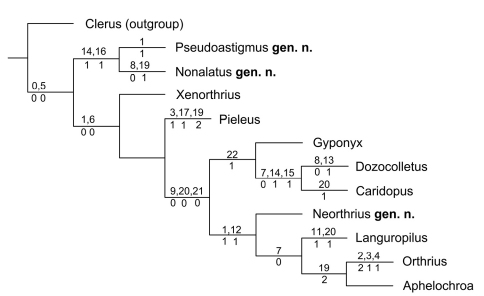
Cladistic tree of the genera of the *Orthrius*-group.

The cladistic analysis resulted in a single most parsimonious tree with a length of 37 steps (Fig. 69). Common to all taxa of the *Orthrius*-group are four mesotarsal pulvilli (char. 0-0) and coarse ommatidial facets (char. 5-0) which distinguishes them from the *Clerus*-series.

**Table 1.** Characters and character states used in the cladistic analysis of the genera.

Character 0	Mesotarsal pulvilli: (0) 4; (1) 3
Character 1	Metatarsal pulvilli: (0) 4; (1) 3; (2) 2
Character 2	Protibial spurs: (0) 2; (1) 1; (2) 0
Character 3	Mesotibial spurs: (0) 2; (1) 1
Character 4	Metatibial spurs: (0) 2; (1) 1
Character 5	Ommatidial facets: (0) coarse; (1) fine
Character 6	Flagellomeres: (0) filiform; (1) dilated
Character 7	Eye’s emargination: (0) absent or weak; (1) conspicuous
Character 8	Eye’s separation: (0) more than two eyes width; (1) between one and two eyes width
Character 9	Gular sutures: (0) convergent to subparallel; (1) subparallel to divergent
Character 10	Gular process: (0) broad; (1) narrow
Character 11	Relation between A2 and A3: (0) A2 < A3; (1) A2 = A3 or A2 > A3
Character 12	Anterior mesosternal process: (0) present; (1) absent
Character 13	Metendosternite, furcal stalk length: (0) normal; (1) very short
Character 14	Metendosternite, furcal arms: (0) normal; (1) acute
Character 15	Metendosternite, furcal stalk base: (0) normal; (1) deeply emarginate
Character 16	Wings: (0) present; (1) absent
Character 17	CuA2: (0) present; (1) absent
Character 18	RP2: (0) present; (1) absent
Character 19	Elytral punctation: (0) with 10 regular striae; (1) with 10 irregular striae; (2) with more than 10 irregular striae
Character 20	Tibial carinae: (0) present; (1) absent
Character 21	Claws: (0) simple; (1) with basal denticle
Character 22	Phallobasic struts: (0) not fused; (1) fused

*Pseudoastigmus* gen. n.and *Nonalatus*gen. n. appear together at the base of the tree. This pair is supported by the acute form of the furcal arms of the metendosternite (char. 14-1) as well as the complete reduction of the hind wings (char. 16-1).

The remaining taxa share the filiform flagellum (char. 6-0). The development of four pulvilli at the metatarsus (char. 1-0) is also synapomorphic at this point, but is reduced to three pulvilli for the cluster of *Neorthrius* gen. n., *Languropilus*, *Orthrius* and *Aphelochroa* (char. 1-1).

These four genera also share the loss of the anterior mesosternal process (char. 12-1). Like in *Dozocolletus* and *Caridopus* the emargination of the eyes is weak or absent (char. 7-0) in *Languropilus*, *Orthrius* and *Aphelochroa*. For this reason, *Neorthrius* adopts a basal position in this group. The monophyly of *Orthrius* and *Aphelochroa* is supported by their elytral punctation (char. 19-2). *Orthrius* differs from all other taxa in this revision in its tibial spur formula which is 0-1-1 (chars. 2-2, 3-1 and 4-1).

The aethiopian genera *Gyponyx*, *Dozocolletus* and *Caridopus* have in common, that the phallobasic struts are fused with the phallobasic apodeme (char. 22-1). The monophyly of *Dozocolletus* and *Caridopus* is well supported by the weak or absent emargination of the eyes (char. 7-0) and similarities of their metendosternites: the furcal arms are acute (char. 14-1) and the furcal stalk base (char. 15-1) is deeply emarginate.

A common ancestor can be assumed for the latter two clusters of genera.This is supported by two synapomorphies: the gular sutures are convergent to parallel (char. 9-0) and the claws are simple (char. 21-0). The presence of the tibial carinae (char. 20-0) also is apomorphic at this node but reduced in *Caridopus* and *Languropilus*. As Solervicens (2007) mentioned, it also may be considered a symplesiomorphy, because it is a common character of the Clerinae.

**Table 2.** Character matrix of 23 adult morphological characters of *Clerus* (outgroup) and genera of the *Orthrius*

Taxa	Characters																						
	0	1	2	3	4	5	6	7	8	9	10	11	12	13	14	15	16	17	18	19	20	21	22
	*Clerus*	1	2	1	0	0	1	1	1	1	1	0	0	0	0	0	0	0	0	0	0	1	1	0
*Aphelochroa*	0	1	1	0	0	0	0	0	1	0	0	0	1	0	0	0	0	0	1	2	0	0	0
*Caridopus*	0	0	1	0	0	0	0	0	1	0	0	0	0	0	1	1	[01]	?	?	0	1	0	1
*Dozocolletus*	0	0	0	0	0	0	0	0	0	0	0	0	0	1	1	1	1	?	?	0	0	0	1
*Gyponyx*	0	0	0	0	0	0	0	1	1	0	0	0	0	0	0	0	0	0	1	0	0	0	1
*Languropilus*	0	1	1	0	0	0	0	0	1	0	0	1	1	0	0	0	0	0	1	0	1	0	?
*Neorthrius*	0	1	1	0	0	0	0	1	1	0	0	0	1	0	0	0	0	0	1	0	0	0	0
*Nonalatus*	0	2	1	0	0	0	1	1	0	1	0	0	0	1	1	1	1	?	?	1	1	1	0
*Orthrius*	0	1	2	1	1	0	0	0	1	0	0	0	1	0	0	0	0	0	1	2	0	0	0
*Pieleus*	0	0	1	1	0	0	0	1	1	1	0	0	0	?	?	?	0	1	1	2	1	1	?
*Pseudoastigmus*	0	1	1	0	0	0	1	1	1	1	0	0	0	?	1	?	1	?	?	0	1	1	0
*Xenorthrius*	0	0	1	0	0	0	0	1	1	1	[01]	0	0	0	0	[01]	0	0	1	0	[01]	1	0

## Supplementary Material

XML Treatment for Aphelochroa

XML Treatment for Caridopus

XML Treatment for Dozocolletus

XML Treatment for Gyponyx

XML Treatment for Languropilus

XML Treatment for Neorthrius

XML Treatment for Nonalatus

XML Treatment for Orthrius

XML Treatment for Pieleus

XML Treatment for Pseudoastigmus

XML Treatment for Xenorthrius
